# Antioxidant Peptide Production Using Keratin from Feather Waste: Effect of Extraction and Thiol Blocking Method

**DOI:** 10.3390/ijms26094149

**Published:** 2025-04-27

**Authors:** Mehrnaz Sheikh Hosseini, Zahra Moosavi-Nejad, Fatemeh Rezaei Sadrabadi, Hamid Hosano

**Affiliations:** 1 Department of Biotechnology, Faculty of Biological Sciences, Alzahra University, Tehran 1993893973, Iran; m.sheikhhosseini@alzahra.ac.ir (M.S.H.); f.rezaei@alzahra.ac.ir (F.R.S.); 2 Biomaterials Department, Institute of Industrial Nanomaterials, Kumamoto University, Kumamoto 860-8555, Japan

**Keywords:** feather keratin, dissolution, thiol-blocking, antioxidant activity, radical scavenging, Fe-chelating

## Abstract

Keratin-made biomaterials, including feathers, are considered a protein-rich bioresource due to their intrinsic properties, including biocompatibility, biodegradability, mechanical resistance, and biological abundance. Beta-keratin exists as an insoluble stringy protein due to the high presence of disulfide cross-links, and as a result, it is mechanically stable and resistant to enzymatic digestion. Because of this, it is not easily decomposed, and this has made the application of feathers difficult. In this study, after dissolving feathers in NaOH, sodium sulfide, and 2-Mercaptoethanol (2-ME), the relative molecular mass of beta-keratin was calculated. Thin-layer chromatography was also used to display proteins with lower molecular weights. The antioxidant activities of the samples were evaluated by Fe-chelating and free radical scavenging tests with 2,2-diphenyl-1-picrylhydrazyl (DPPH). To investigate the effect of blocking thiol groups on the antioxidant activity of dissolved keratin, iodoacetamide and H_2_O_2_ were used. According to the three methods—(A) sodium hydroxide, (B) sodium sulfide, and (C) urea and 2-ME—used to extract and dissolve the feathers, method C caused the least change in the chemical structure of keratin molecules. Method A destroyed the primary structure of keratin and drastically reduced its molecular mass, but method B caused a drastic increase in the molecular mass from 9.6 kDa to higher masses, due to intermolecular bonds. For the keratin molecules dissolved by method C, the Fe-chelating activity was 93.18% and free radical scavenging was 77.45%. Blocking the thiol group with iodoacetamide initially reduced the free radical scavenging activity with DPPH by 42%, but blocking it with H_2_O_2_ did not affect this activity. Also, blocking of the thiol group did not initially affect Fe-chelating activity and free radical scavenging activity. After a kinetic study of the activities, an interesting observation was that both blocking agents had negative effects on radical scavenging activity, but had positive effects on Fe-chelating activity. This indicates the complexity of the role of disulfide bonds in keratin’s antioxidant behavior types. According to the observed antioxidant activities, it can be expected that beta-keratin extracted from chicken feathers is a suitable candidate for application in industrial, pharmaceutical, and health applications.

## 1. Introduction

Bioactive peptides (BPs) are obtained from natural sources, and include small amino acid fragments that can cause physical and chemical changes in the body’s natural processes [1]. These physicochemical effects are related to their amino acid composition, sequence, and molecular weight [2,3]. The presence of hydrophobic amino acids enhances the radical removal activity and augments the antioxidant activity of peptides, giving them great potential for a wide range of applications [4]. On the other hand, bioactive peptides have fewer side effects and immunogenicity than large protein-based biopharmaceutical drugs [5]. There are many reports indicating that peptides obtained from protein hydrolysis may show bioactive activity [6,7,8,9]. Currently, more than 4400 different BPs are reported in the BIOPEP-UWM database, which documents their antimicrobial, antioxidant, anti-inflammatory, and other bioactivities [10,11].

Free radicals exert detrimental effects on both food and biological systems through the process of oxidation. Specifically, they can diminish the quality of food and impair cell membrane function by impacting membrane lipids [12,13]. Additionally, free radicals have been associated with the development of diabetes, coronary heart disease [14], arteriosclerosis [15], high blood pressure, cancer [16], Alzheimer’s [17], Parkinson’s [18], DNA damage, and aging [19]. On the other hand, antioxidant substances can promote the healing of both infectious and non-infectious wounds by mitigating the harm inflicted by free radicals [20]. Given that certain antioxidants can enhance immune system function [21], there is currently a growing demand for natural antioxidants, due to their long-term immunogenicity and a decrease in the consumption of synthetic antioxidants [22]. Researchers have demonstrated that several hydrolyzed proteins derived from both plant and animal sources exhibit antioxidant activity by effectively eliminating free radicals, donating electrons, or chelating metal ions [23,24,25].

Due to the growing utilization of animal by-products, numerous researchers have commenced investigating animal peptides as a potential source of natural antioxidants [26,27,28]. Efforts are being made globally to enhance the utilization of biological products. Given the constraints posed by limited natural resources, particular emphasis has been placed on utilizing industrial by-products as renewable resources. One such example of a by-product from poultry farms is chicken feathers, which are widely accessible and economically viable, constituting over 10% of the bird’s total weight [29]. Chicken feathers consist of 90% keratin, and contain significant quantities of amino acids, such as glycine, alanine, serine, cysteine, arginine, phenylalanine, and valine. Consequently, utilizing keratin-rich waste materials can be an efficient method for acquiring bioactive peptides [30]. In 2014, Fontoura and colleagues successfully conducted enzymatic digestion of the proteins in chicken feathers, and subsequently investigated their antioxidant and antihypertensive properties [31]. In 2015, Sundaram and colleagues dissolved chicken feathers using sodium hydroxide (NaOH), and then ethanol and glutaraldehyde, to generate keratin nanoparticles possessing antioxidant and antimicrobial characteristics [32].

In this study, an attempt was made to solubilize keratin, while preserving the integrity of its molecular structure. This approach not only reduces costs and processes, but also results in a well-defined and pure keratin solution, thereby minimizing the risk of side effects such as immune system stimulation caused by unknown compounds. The resulting keratin solution does not contain diverse peptides of unknown length or ambiguous compounds, and the number of components in this solution is limited to the natural components present in the full structure. Furthermore, we clearly demonstrate in this study that the intact keratin molecule has significant antioxidant properties (both free radical scavenging and Fe-chelating), and that the effects of chemical agents, including various chemical solvents or biological agents, such as microbial degradation and the action of proteolytic enzymes, should be avoided in order to maintain these intrinsic antioxidant activities at their maximum initial value. Additionally, attempts were made to block the thiol groups present in the keratin structure to prevent the formation of disulfide bonds and ensure that the keratin molecule did not revert to an insoluble state.

## 2. Results

### 2.1. Feather Dissolution

**Figure 1:**
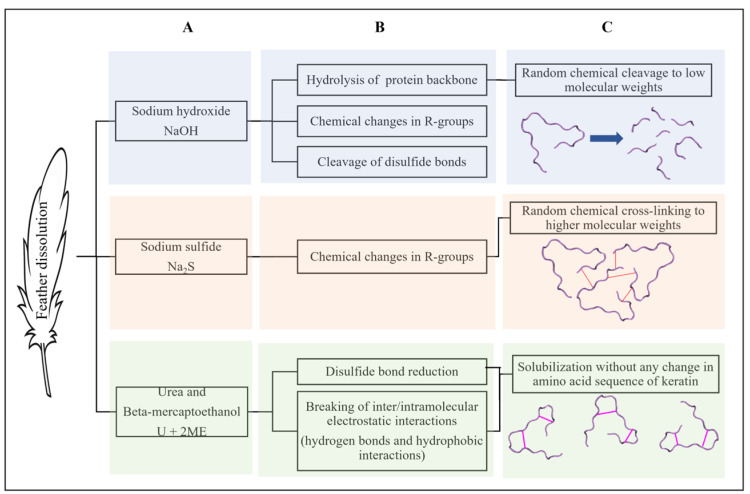
Chemical methods of feather dissolution in this article. (**A**) Chemical agents. (**B**) Effects of agents on keratin molecule. (**C**) Final results of physicochemical characterization of keratin molecules.

The results of feather dissolution showed that in all three methods (NaOH, Na_2_S, and urea with 2-ME), the feather was dissolved well (Figure 1). Also, the solutions after chemical treatment in all three methods were clear, and no turbidity was observed. Considering that after dialysis, small peptides and amino acids resulting from hydrolysis or chemical breakdown are removed from the solution, it was expected that only chemical molecules that are the size of keratin or larger would remain in the solution.

### 2.2. Characterization of Three Dialysates

**Figure 2:**
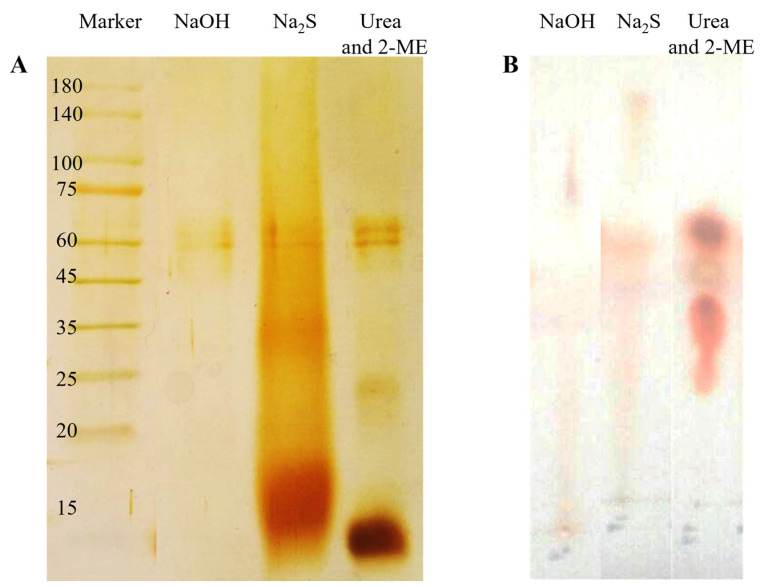
Electrophoretic pattern (**A**) and thin-layer chromatogram (**B**) of solution of keratin solubilized by sodium hydroxide (NaOH), sodium sulfide (Na_2_S), and urea and 2-ME. In Tricine SDS-PAGE, gel was stained using AgNO_3_. Chromatogram was stained using ninhydrin.

The results of gel electrophoresis and thin-layer chromatography were compared for samples obtained from all three methods, in order to choose the best method for future experiments. To ensure the integrity of the beta-keratin protein structure during the dissolving process, the resulting polypeptide composition was checked using the Tricine SDS-PAGE method (Figure 2), after dissolving the feather through the three methods mentioned above. Figure 2A shows the results of the beta-keratin solution prepared with urea and 2-ME. Three separate bands were observed in the gel, with the lowest band having the highest concentration, indicating beta-keratin, and two bands with higher molecular mass and lower concentration, suggesting the presence of other proteins in the feather structure. The presence of specific and separate bands in the gel can be attributed to the performance of urea and 2-ME, as this method does not break peptide bonds. However, the sample resulting from dissolving the feather with sodium sulfide solution (Figure 2A) showed a smear instead of specific bands in the gel. Both the smear and the bold band were shifted towards higher molecular masses than the beta-keratin band, indicating non-specific chemical cross-linking between beta-keratin molecules, caused by sodium sulfide. On the other hand, dissolving beta-keratin with NaOH resulted in the opposite outcome compared to the previous methods. The sample dissolved in NaOH did not show any distinct bands in the gel (Figure 2A), indicating the creation of amino acids and small peptides due to the action of NaOH, most of which were removed from the gel during dialysis. Even if they did not pass through the dialysis tube, they were rejected due to the small size of the gel electrophoresis pores, resulting in no bands being observed in the gel.

The results of thin-layer chromatography (TLC) confirmed the findings of gel electrophoresis. As shown in Figure 2B, the sample dissolved in urea and 2-ME produced a specific spot on the chromatography paper (Figure 2B), while the samples dissolved in sodium sulfide and NaOH did not create specific spots, and appeared as smears (Figure 2B). The results of TLC and gel electrophoresis were consistent for the sample dissolved in sodium sulfide, with no bands observed in gel electrophoresis, but a smear seen in TLC. This can be attributed to the ability of TLC to detect amino acids and peptides with low molecular weight. Based on the results of Tricine-SDS-PAGE and TLC, the keratin solution obtained by dissolving the feather with urea and 2-ME was chosen for further steps.

### 2.3. Biochemical Characterization of Feather Keratin

**Figure 3:**
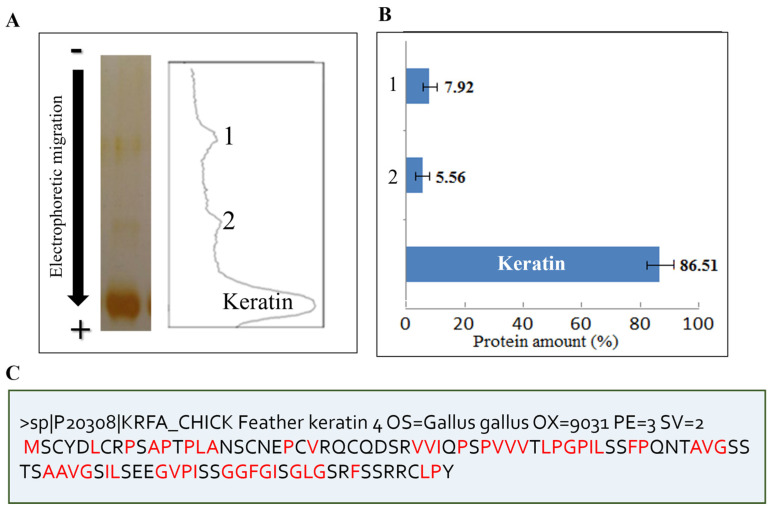
Characterization of keratin solution, solubilized by urea and 2-ME. (**A**) Electrophoretic pattern in tricine-SDS-PAGE and its scanning analysis, and (**B**) quantification of gel scan by ImageJ software. (**C**) Amino acid sequence of chicken feather keratin.

To determine the molecular mass of the dissolved beta-keratin protein, Tricine-SDS-PAGE electrophoresis was conducted (as shown in Figure 2), and a standard diagram for molecular mass determination was created. According to the equation of the standard curve, the molecular mass of the keratin protein was determined to be 9.62 kDa, while the observed impurity molecular masses were 24.21 and 63.15 kDa. Therefore, it can be concluded that keratin protein naturally exists as a peptide with a molecular weight of 9.6 kDa. Previous research has also reported that chicken feather proteins are in the form of peptides [28]. Analysis of protein bands in the electrophoresis gels revealed that 86.51% of the protein was beta-keratin, while the remaining 13.48% consisted of impurities with a higher molecular mass (Figure 3A,B). However, it should be noted that these calculations were based on dialyzed samples, and some small peptides may have been removed from the keratin solution during dialysis. Consequently, the actual percentage of beta-keratin is expected to be higher than the aforementioned value.

The sequence of Gallus gallus (Chicken) feather keratin was obtained from Uniprot with the code P20308. This protein has 98 amino acids, with a calculated molecular mass of 10.4 kDa. As shown in the sequence of chicken feather keratin in Figure 3C, chicken feather keratin contains the amino acids glycine, proline, phenylalanine, leucine, isoleucine, alanine, valine, and methionine, which are considered hydrophobic amino acids; these are equivalent to 51.02% of the keratin sequence.

Determining the concentration of beta-keratin protein in solution is essential for various biochemical calculations. To determine the protein concentration, a standard curve was constructed using Bradford’s method. The concentration of the beta-keratin protein solution was then calculated using the equation derived from the standard curve. The concentration of the beta-keratin solution was found to be 2.76 ± 0.65 mg/mL. Soluble keratin has a free radical scavenging activity of 93.59% and an Fe-chelating activity of 93.17%.

### 2.4. Effect of Blocking Agents on Antioxidant Activity

**Figure 4:**
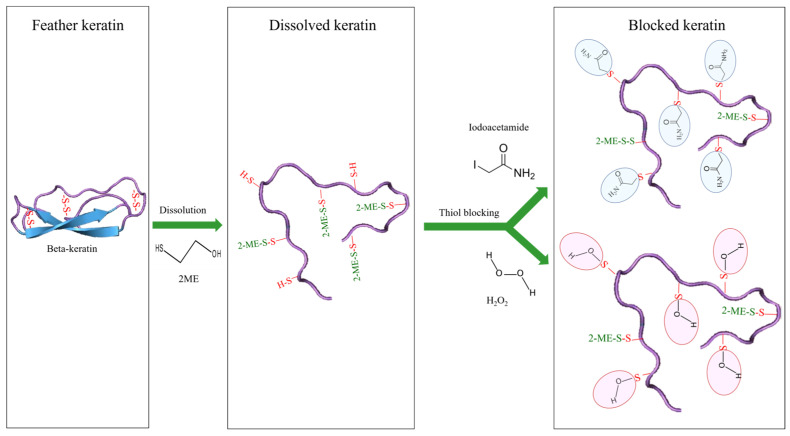
Chemical status of thiol groups belongs to cysteine residues of keratin molecules. In feathers, keratin solution and blocked keratin solution by both iodoacetamide and H_2_O_2_ methods.

In this study, after the disulfide bonds were broken by urea and 2-ME, the free thiol groups were blocked by iodoacetamide and H_2_O_2_ (Figure 4).

### 2.5. Radical Scavenging Activity

**Figure 5:**
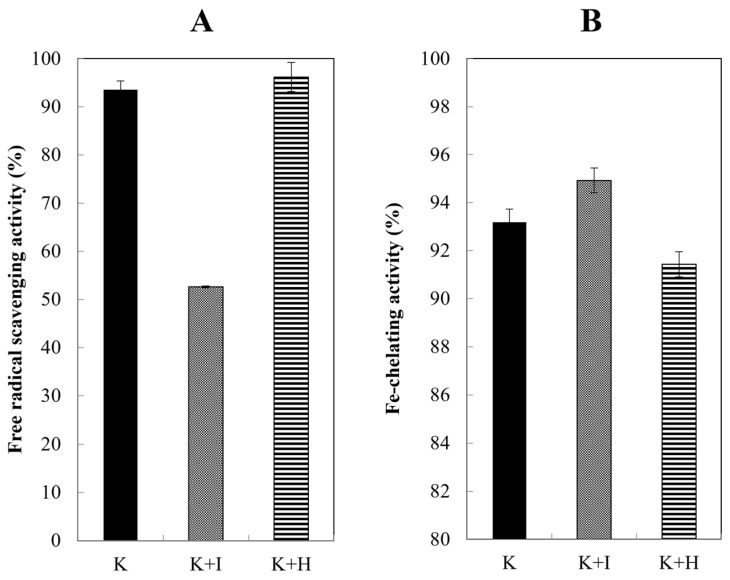
Antioxidant activity of keratin solution. (**A**) Free radical scavenging activity. (**B**) Fe-chelating activity for keratin molecule with unblocked thiol groups (K), keratin blocked by iodoacetamide (K + I), and keratin blocked by H_2_O_2_ (K + H).

According to Figure 5A, it was observed that the addition of H_2_O_2_ to the keratin solution did not result in a significant alteration in the rate of free radical inhibition. However, the inclusion of iodoacetamide led to a reduction of approximately 42% in inhibitory activity, and negatively impacted the sample’s overall activity.

### 2.6. Fe-Chelating Activity

In this study, the antioxidant property was also investigated by measuring the ability to chelate iron metal. Based on Figure 5B, all three samples showed chelation of a substantial amount of iron metal, and there were no significant differences. Interestingly, unlike the DPPH test, blocking did not reduce the chelating properties. Thus, it appears that the iron chelating activity is carried out by groups other than the thiol group.

### 2.7. Kinetics Studies

#### 2.7.1. Kinetics of Free Radical Scavenging

**Figure 6:**
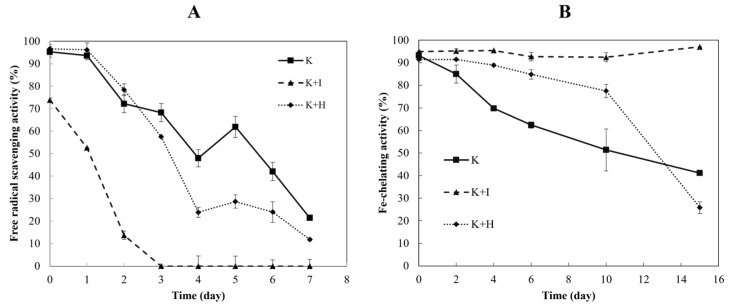
Kinetics of antioxidant activity of keratin solution. (**A**) Free radical scavenging activity. (**B**) Fe-chelating activity for keratin molecule with unblocked thiol groups (K), keratin blocked by iodoacetamide (K + I), and keratin blocked by H_2_O_2_ (K + H).

After observing the inhibitory action of the keratin solution on free radicals, the stability of this observed activity was investigated by studying the kinetics of the solution. Figure 6A reveals that the ability of the keratin solution to remove DPPH free radicals diminished over time. Furthermore, iodoacetamide was identified as a factor that negatively affected the performance of the keratin solution, with the best performance observed when the solution was unblocked.

#### 2.7.2. Kinetics of Fe-Chelating

Figure 6B shows the high Fe-chelation activity of the samples. This feature is more stable over time compared to the free radical scavenging activity. As depicted in Figure 6A, the sample blocked by iodoacetamide continued to exhibit maximum activity even after two weeks. Hence, blocking had a beneficial impact on the Fe-chelating property of the samples.

### 2.8. Comparison of Rate of Inactivation

According to Table 1, the use of blocking agents negatively affected the k inactivation caused by free radical scavenging activity. When thiol groups were blocked with iodoacetamide, the k inactivation due to free radical scavenging increased by approximately four times, and H_2_O_2_ increased by about two times.

Blocking had a positive impact on the rate of Fe-chelating activity loss in the keratin solution. The keratin solution had an inactivation constant of 0.024, and when thiol groups in the keratin solution were blocked with iodoacetamide, the inactivation constant decreased to 0.0002. This indicates that the samples blocked with iodoacetamide experienced a slower rate of activity loss. When H_2_O_2_ was used for blocking, the k inactivation reached 0.033. Therefore, blocking with H_2_O_2_ does not affect the rate of deactivation of chelating activity.

## 3. Discussion

Beta-keratin is a type of water-insoluble protein, making it difficult to decompose. Due to the presence of numerous disulfide bonds in its structure, non-ruminant animals can only decompose it in small amounts. Moreover, beta-keratin is resistant to enzymatic hydrolysis, posing a challenge for its application. Several scientists have conducted research in this field. In 1970, Nagai and Nishikawa applied different concentrations of NaOH to chicken feathers, and analyzed the resulting amino acids [33]. Steiner and colleagues dissolved chicken feathers using NaOH and H_3_PO_4_ [34]. Papadopoulos explained that varying amounts of NaOH or maxatase enzyme can break the disulfide bonds, leading to subsequent enzymatic hydrolysis [35]. In 2002, Kim exposed chicken feathers to NaOH and pepsin enzyme, exploring the time parameters of their effects under different conditions [36]. Additionally, some scientists have investigated the effects of conditions such as high temperatures and pressure [37,38], as well as acid hydrolysis [39], on the dissolution of chicken feathers. Moreover, Schrooyen and colleagues used a solution of urea and 2-ME to restore the links in full keratin [40]. Mokrejs also used urea and 2-ME to dissolve chicken feathers, but employed SDS to prevent the re-formation of disulfide bonds after dialysis [41]. Fontoura and colleagues produced feather hydrolysate using KH_2_PO_4_, NaCl, and CaCl_2_ [31]. Recently, Pakdel and colleagues dissolved chicken feather keratin using urea and 2-ME [42]. Although various methods have been employed to address the solvation problem of feathers, each method has its drawbacks: (a) high pressure and temperature can damage certain amino acids and alter the amino acid composition of the protein, and (b) acid and alkaline hydrolysis can break the backbone of the polypeptide chain and disrupt the protein’s primary structure, resulting in a loss of essential amino acids and changes in others. Consequently, some researchers have turned to milder substances for dissolving beta-keratin. For this reason, the use of harsh physicochemical methods to dissolve feather keratin is undesirable, especially because protein bioactivities, such as antioxidant activity, are highly dependent on the health and integrity of the amino acid side chains from which proteins are made. Therefore, among the methods used to dissolve feathers, the method using urea and 2-ME, which cause the least chemical change in the structure of the keratin molecule, is very suitable.

Antioxidant properties depend on the structural properties of peptides or proteins, including their molecular weight, hydrophobicity, and amino acid sequence [43]. Studies show that low-molecular-weight peptides show effective antioxidant properties [44]. Therefore, the protein synthesized in this study with a low molecular mass (9.62 kDa) and a hydrophobicity of 51.02% is suitable, and is expected to have antioxidant properties, which is confirmed by the findings in Table 1. In this study, the sequence of chicken feather keratin and its hydrophobic amino acids were investigated, and the hydrophobicity of keratin was stated as one of the factors affecting the antioxidant properties of chicken feather keratin. This finding is in accordance with the study of Pakdel and colleagues, who mentioned the hydrophobicity of keratin [42]. It has also been reported in other studies that the inner surface of each beta-sheet of feather keratin is densely filled with hydrophobic residues [45]. In addition, in the turns between the two strands of the beta-sheet of keratin, a highly conserved hydrophobic sequence is found, which includes leucine/isoleucine-proline-glycine-proline, and causes hydrophobic patches on the surface of keratin molecules [46].

In the present study, different methods for the dissolution of chicken feathers were compared, and the antioxidant properties of the dissolved keratins were assessed using Fe-chelation and DPPH (2,2-diphenyl-1-picrylhydrazyl) free radical inhibition assays. Additionally, the effect of agents blocking thiol groups on the antioxidant properties was also considered. Previous studies have primarily focused on assessing the antioxidant activity of protein samples obtained through dissolution, with only a few studies investigating the antioxidant properties of feathers using bacterial and chemical hydrolysis methods.

Alahyaribeik and colleagues (2020) conducted a study on the antioxidant activity of feathers, and found that feather proteins reduced with sodium sulfite exhibited a high antioxidant activity [47]. In the same year, Bouhamed and colleagues demonstrated that acid-hydrolyzed feather protein possessed antioxidant activity [37]. Fountra and colleagues (2019) confirmed the antioxidant activity of feather hydrolysate using ABTS and TRAP assays [48]. Fontoura and colleagues (2014) hydrolyzed chicken feathers using bacterial digestion, and evaluated the biological properties of the resulting proteins and peptides [31]. Sundaram and colleagues (2015) successfully produced nanoparticles from chicken feathers using chemical methods, and investigated the antioxidant properties of the nanoparticles during their experiments [32].

Keratin shows antioxidant activity in two ways: firstly, due to the presence of hydrophobic amino acids (keratin has 51.02% hydrophobicity), according to the articles published in this field (mentioned above); and secondly, due to the presence of a disulfide bond, which, depending on the type of antioxidant activity desired, has a dual effect.

One of the conventional methods to prevent the reversibility of disulfide bonds between free thiol groups is to neutralize them by creating chemical substitutions. When 2-ME and urea are removed during dialysis, the exposed thiol groups have the potential to re-form disulfide bonds, leading to the accumulation of protein particles. To address this issue, it is common to use substances like iodoacetamide, which can block thiol groups. In a study conducted by Schrooyen and colleagues in 2000, feather dissolution was achieved using urea and 2-ME, and iodoacetamide was used to block thiol groups and prevent the re-formation of disulfide bonds [40]. Additionally, hydrogen peroxide can also react with free thiol groups. In this research, H_2_O_2_ was used as an agent to block thiol groups, along with iodoacetamide. It is possible that the blocking of thiol groups resulted in the creation of new chemical substitutions in the thiol group, thereby improving chelating activity.

If it is possible to prevent protein accumulation without blocking thiol groups, better results can be obtained from antioxidant tests. This is because preventing protein clumping reduces the reduction in keratin’s antioxidant properties. It is important to note that the thiol group in cysteine is responsible for keratin’s antioxidant properties, and blocking thiol groups reduces this property.

## 4. Materials and Methods

DPPH was obtained from Sigma-Aldrich (Louis, MO, USA). All other materials were purchased from Merck (Darmstadt, Germany), and were of analytical grade. White chicken feathers were collected from the Sepahan Morgh slaughterhouse in Isfahan city, Iran. Initially, the feathers were washed using water pressure, and then were washed twice with detergent. Subsequently, they were rinsed eight times with tap water and three times with distilled water. The feathers were then dried at 40 °C for 48 h in an incubator. The dried feathers were chopped into small pieces with a maximum size of 1–2 mm to be used for dissolving.

### 4.1. Dissolution of Feathers

Three methods were used for extracting keratin from feather waste. Feathers were dissolved in each solution, and for each method, the resulting solution after dialysis, referred to as “dialysate”, was then used for subsequent experiments.

#### 4.1.1. Dissolution Using Sodium Hydroxide

A solution of sodium hydroxide (10 mL, 3N) was added to two grams of chopped feathers. The resulting solution was then filtered and centrifuged at 10,000× *g* for 30 min [37,38]. Subsequently, the supernatant was dialyzed (cutoff of 12–15 kDa) against distilled water for 72 h at 4 °C.

#### 4.1.2. Dissolution Using Sodium Sulfide

Chopped feathers weighing 0.5 g were dissolved in 20 mL of 0.5 M sodium sulfide solution. The resulting protein solution was filtered and centrifuged at 8000× *g* for 15 min. The supernatant was then dialyzed against distilled water for 72 h at 4 °C, using a dialysis tube with a molecular weight cutoff (MWCO) of 12–15 kDa [40].

#### 4.1.3. Dissolution Using Urea and 2-Mercaptoethanol

A Tris buffer solution with a concentration of 25 mM (375 mL) and a pH of 8.5 was prepared. Urea and 2-Mercaptoethanol (2-ME) were added to the solution. Three grams of chopped feathers were then added to the solution. The resulting protein solution was filtered using filter paper, and centrifuged at a speed of 10,000× *g* for 30 min. The liquid portion above the sediment, known as the supernatant, was separated. The supernatant was subjected to dialysis against distilled water at a temperature of 4 °C for 72 h. Dialysis was performed using a dialysis tube with a molecular weight cutoff (MWCO) of 12–15 kDa [40]. The dialysate was called “keratin solution”.

### 4.2. Protein Characterization

#### 4.2.1. Tricine-SDS-PAGE Analysis

Tricine Sodium dodecyl sulfate-polyacrylamide gel electrophoresis (Tricine-SDS-PAGE) was used to determine the molecular mass and purity of keratin dissolved in urea and 2-ME. This method is often used for peptides and proteins with a low molecular mass [49]. Electrophoresis was performed in a vertical slab gel unit (Akhtrian (Tehran, Iran)), with a gel thickness of 1.5 mm, for three gels: separating (16.5%), spacer (10%), and stacking (4%). For tank buffers, two different buffers were used, including an anode buffer of pH 8.9, 0.2 M Tris-HCl, and a cathode buffer of 0.1 M Tricine, pH 8.25, 0.1 M Tris, 0.1% sodium dodecyl sulfate (SDS). Sample volumes of 20 μL were added to wells of the gel, and electrophoresis was performed at a voltage of 100 V, until the bromophenol blue band reached the end of the separating gel. After electrophoresis, the gel was placed in the fixing solution, staining solution, and destaining solution for 1 h, respectively.

#### 4.2.2. Thin-Layer Chromatography (TLC)

Volumes of 10 µL of each dialysate sample and the control sample were placed at 1 cm intervals on Silicagel F254 paper. Once the stains were completely dried, the TLC paper was placed in an electrophoresis tank. The solvent used consisted of butanol, acetic acid, and water in a ratio of 1:1:1.5. When the solvent reached one centimeter from the upper edge of the chromatography paper, the paper was removed from the tank and placed in an oven at a temperature of 100 °C to dry the solvent. To observe the protein and peptide spots, ninhydrin solution was sprayed onto the paper. Finally, the chromatography paper was dried in an oven [38].

### 4.3. Protein Quantification

The Bradford method was utilized to determine the protein concentration [50]. To quantify the protein content in the keratin solution, 50 µL of the keratin-containing solution was mixed with 625 µL of Bradford solution. After a 2 min incubation period, the absorbance of the sample was measured at a wavelength of 595 nm.

### 4.4. Blocking of Free Thiol Groups with Iodoacetamide

To block thiol groups using iodoacetamide, a keratin solution was prepared using “the urea and 2-ME” method and mixed with iodoacetamide (0.021 M). The mixture was then placed on a stirrer in a dark environment. Subsequently, the prepared solution was dialyzed against water for 40 h, at a temperature of 15 °C [51].

#### 4.4.1. Blocking of Free Thiol Groups with H_2_O_2_

To block thiol groups, a solution of H_2_O_2_ (0.11M) was added to the keratin solution [51].

#### 4.4.2. Measurement of Free Radical Scavenging Activity

To each of the keratin samples (100 µL), 100 µL of methanol and 1 mL of 0.05 mM DPPH solution were added, and the mixture was incubated on ice in a dark environment for 90 min. The absorbance of the samples was measured at a wavelength of 517 nm [52]. The activity of removing free radicals was calculated using Formula (1):

Radical scavenging activity = ((Ac − As)/Ac) × 100 Control absorption (methanol) = Ac Sample absorption = As(1)

### 4.5. Measurement of Fe-Chelating Activity

A 1 mL sample of keratin was added to a 17 µL solution of 2 mM FeCl_2_. The reaction was initiated by adding 70 µL of ferrozine solution. The mixture was then incubated at room temperature for 10 min. The absorbance of the samples was measured at a wavelength of 562 nm. To ensure the accuracy of the test, a positive control of EDTA (20 mg/mL) was used [53]. The Fe-chelating activity was calculated using Formula (2):

Fe-chelating activity %= ((Ac − As)/Ac) ×100 Ac = absorbance control (EDTA) As = absorbance of keratin sample(2)

### 4.6. Kinetics of Antioxidant Activity

A keratin solution was prepared for daily testing. The required volume was taken from the solution prepared on the first day. The iron chelation test and DPPH free radical inhibition test were then performed. These tests were conducted until the activity reached its lowest value, or until changes ceased. To compare the rate of inactivation of the samples, the logarithm of the activity percentage was calculated each day. Using these data, a logarithm diagram was created to show the deactivation percentage against the heating time. The deactivation rate constant was determined by calculating the slope of the line obtained from the diagram.

### 4.7. Bioinformatic Analysis

The Uniprot website was used to study the sequence of chicken feather keratin (P20308) (https://www.uniprot.org).

### 4.8. Data Analysis

All experiments were performed three times, and statistical analyses were performed using Excel 2016 software. To determine the concentration of keratin and the presence of impurities in the protein solution, the electrophoresis gel was analyzed using gel scanning software (ImageJ (version 1.53 t)), available at http://rsb.info.nih.gov/ij/ (accessed on 3 March 2025).

## 5. Conclusions

In the present study, we investigated the proteins and bioactive peptides obtained from the dissolution of chicken feathers. We found that approximately 86.5% of the protein composition of the dissolved and dialyzed feathers was beta-keratin, with a relative molecular mass of 9.6 kDa. In terms of the biological activities that determine the potential applications of beta-keratin, this protein exhibited a high amount of two different antioxidant bioactivities: Fe-chelating (93.18%) and free radical scavenging (77.45%). Due to the high protein content of chicken feathers and their observed biological activities, they are a highly suitable option in the farming industry as a supplement for livestock and poultry feed. Additionally, we examined the changes in the two antioxidant activities resulting from the blocking of beta-keratin soluble thiol groups using iodoacetamide (a common substance used for blocking thiol groups) and H_2_O_2_ (an uncommon substance for this purpose). We found that blocking thiol groups by creating new substitutions and changing their chemical properties affected the antioxidant activities of the samples.

Comparing the opening and blocking of the disulfide bond of keratin with two different methods showed that the disulfide bond definitely has an effect on antioxidant activity, and is a determining factor. However, depending on the type of antioxidant behavior, it may have a positive or negative effect. In other words, one antioxidant behavior, radical scavenging activity, has a negative effect in that by removing the disulfide bond, the antioxidant activity decreases. Another antioxidant behavior, Fe-chelating activity, seems to have a positive effect in that by breaking or blocking the disulfide bond, the antioxidant activity increases. This indicates the complexity of the role of the disulfide bond in the types of antioxidant behaviors for keratin.

## Figures and Tables

**Table 1:** Relative changes in rate of inactivation (K_in_) of blocked and non-block thiol-containing keratin molecules.

Thiol Blocking Agents	K_in_ of Free Radical Scavenging Activity (%)	K_in_ of Fe-Chelating Activity (%)
-	0.09	0.024
Iodoacetamide	0.37	0.0002
H_2_O_2_	0.19	0.033

## Data Availability

The data presented in this study are available on request from the corresponding author.
